# Oral Microbiota and Carcinogenesis: Exploring the Systemic Impact of Oral Pathogens

**DOI:** 10.3390/pathogens14121233

**Published:** 2025-12-03

**Authors:** Nađa Nikolić, Ana Pucar, Uroš Tomić, Sanja Petrović, Đorđe Mihailović, Aleksandar Jovanović, Milena Radunović

**Affiliations:** 1 School of Dental Medicine, University of Belgrade, 11000 Belgrade, Serbia; nadja.nikolic@stomf.bg.ac.rs (N.N.);; 2 Department of Dentistry, Faculty of Medicine, University of Priština with Temporary Headquarters in Kosovska Mitrovica, 10000 Kosovska Mitrovica, Serbia; 3 Clinic of Urology, University Clinical Center of Serbia, 11000 Belgrade, Serbia

**Keywords:** oral microbiome, dysbiosis, *Porphyromonas gingivalis*, *Fusobacterium nucleatum*, *Treponema denticola*, carcinogenesis, oral–gut axis

## Abstract

For decades, cancer risk has been explained mainly by local factors. However, emerging evidence shows that the oral microbiome acts as a systemic modifier of oncogenesis well beyond the head and neck. This review synthesizes clinical and mechanistic data linking dysbiotic oral communities, especially *Porphyromonas gingivalis*, *Fusobacterium nucleatum*, and *Treponema denticola*, to malignancies across gastrointestinal, respiratory, hepatobiliary, pancreatic, breast, and urogenital systems. We summarize organ-specific associations from saliva, tissue, and stool studies, noting the recurrent enrichment of oral taxa in tumor and peri-tumoral niches of oral, esophageal, gastric, colorectal, lung, pancreatic, liver, bladder, cervical, and breast cancers. Convergent mechanisms include the following: (i) persistent inflammation (lypopolysacharide, gingipains, cytolysins, and collagenases); (ii) direct genotoxicity (acetaldehyde, nitrosation, and CDT); (iii) immune evasion/suppression (TLR/NLR signaling, MDSC recruitment, TAN/TAM polarization, and TIGIT/CEACAM1 checkpoints); and (iv) epigenetic/signaling rewiring (NF-κB, MAPK/ERK, PI3K/AKT, JAK/STAT, WNT/β-catenin, Notch, COX-2, and CpG hypermethylation). Plausible dissemination along an oral–gut–systemic axis, hematogenous, lymphatic, microaspiration, and direct mucosal transfer enables distal effects. While causality is not yet definitive, cumulative data support oral dysbiosis as a clinically relevant cofactor, motivating biomarker-based risk stratification, saliva/stool assays for early detection, and microbiome-targeted interventions (periodontal care, antimicrobials, probiotics, and microbiota modulation) alongside conventional cancer control.

## 1. Introduction

Over several decades, the story about cancer development that has been told was centered around local risk factors, with the mouth mostly viewed through the narrow lens of head and neck cancers. But in recent years, that picture has started to shift and quite dramatically. We are now seeing compelling evidence that the oral microbiome may play a far more expansive role, potentially influencing the development and progression of cancers in tissues far beyond the oral cavity.

The mouth, as a complex microbial ecosystem, hosts a diverse mix of bacteria, some of which support oral health, while others, like *Porphyromonas gingivalis* (*P. gingivalis*), *Aggregatibacter actinomycetemcomitans* (*A. actinomycetemcomitans*), and *Fusobacterium nucleatum* (*F. nucleatum*) group [1], are far more sinister. These pathogens produce aggressive compounds, i.e., lipopolysaccharides (LPSs), cytolysins, and collagenases, leading to chronic inflammation and DNA damage, and helping microbes evade immune defenses. In other words, they tick all the boxes of what drives cancer at the cellular level [2,3,4].

What is truly fascinating, and a little unsettling, is the way these oral bacteria seem to affect the body far beyond the mouth. This so-called oral–gut–systemic axis acts like a superhighway for microbial signals, allowing bacteria and their byproducts to travel and exert influence elsewhere. While one of the better-known theories focuses on how bacteria from diseased gums enter the bloodstream and cause systemic effects, new research points to another route: the gut. It appears that certain oral pathogens can disrupt the gut microbiota balance, contributing to broader immune dysregulation and chronic disease [5]. That opens the door for oral microbes to shape cancer risk in tissues that were once thought to be out of their reach.

The bacterial translocation is made possible through several potential routes, including the following:

- Through the bloodstream, i.e., hematogenous dissemination—inflamed periodontal tissues act as an entry point, letting bacteria into circulation. In fact, some studies have detected oral bacteria inside the walls of blood vessels in atherosclerotic plaques [6,7].
- Via the lymphatic system—from the oral mucosa, pathogens may access distant sites by utilizing the lymphatic channels [8].
- Through the digestive tract—oral–gut translocation can occur via continuous swallowing of saliva, as approximately 1–2 L of saliva containing up to 108 oral bacteria per milliliter are swallowed daily, potentially enabling microbial migration to the gastrointestinal tract [9].
- Through direct contact—poor oral hygiene combined with oral–genital contact can allow pathogens to colonize parts of the urogenital tract [10].

Establishing a direct cause-and-effect relationship between oral bacteria and specific cancers remains a major scientific challenge. The nature of cancer development is complex. It rarely has a single trigger, and both genetic predispositions and environmental exposures blur the picture. Still, study by study, we are inching closer to understanding the molecular mechanisms by which specific oral bacteria might promote carcinogenesis. From manipulating immune signaling and damaging epithelial barriers to rewiring host cell communication, their toolkit is disturbingly sophisticated, and this review will try to explain the intricate mechanisms by which oral bacteria might contribute to carcinogenesis.

Although the connection between oral microbes and head and neck cancers is already well-documented and perhaps unsurprising given the anatomical proximity, emerging data are drawing attention to possible links with cancers in areas traditionally considered “sterile,” such as the liver, lungs, breasts, and reproductive organs. While the direct bacterial colonization of these sites is unlikely, what seems more plausible is a kind of indirect orchestration: chronic inflammation, systemic immune modulation, and a disrupted microbiome all converge to make the environment more favorable for tumor formation.

For some oral pathogens a large body of consistent experimental, clinical, and meta-analytical evidence outlines their mechanisms in cancer development. For others, the associations are still at the level of preliminary evidence, lacking proper validation in large clinical cohorts. Some oral microorganisms have been identified in the metagenomic analyses of various tumors but without clear causal evidence or a mechanistic link supporting a direct role in carcinogenesis. Owing to that, in the following sections, we provide a comprehensive summary of the available literature on the role of oral microorganisms in the carcinogenesis of multiple organ systems.

## 2. Organ and System-Specific Influences

### 2.1. Oro-Gastrointestinal Cancers

#### 2.1.1. Oral Cancer

Oral cancer ranks among the six most commonly diagnosed malignancies worldwide, with oral squamous cell carcinoma (OSCC) accounting for roughly 90% of all cases [11]. Oral carcinogenesis is a highly complex, multistep process driven by both genetic and epigenetic changes in oral keratinocytes, often triggered by environmental exposures collectively referred to as the exposome [12].

Established risk factors for OSCC include tobacco use (both smoking and chewing), chronic alcohol consumption, poor dietary habits, and, notably, inadequate oral hygiene [13]. In recent years, the potential role of periodontal disease, particularly chronic periodontitis, has been linked to elevated cancer risk [14]. This elevated susceptibility to cancer development in individuals with periodontitis may be linked to oral bacterial composition shifts observed in individuals with periodontitis. Namely, the disruption of microbial balance, where pathogenic species become more dominant, could contribute to a proinflammatory and potentially pro-carcinogenic environment in the oral cavity [15].

A growing body of the literature supports the frequent detection of specific bacterial species in patients with OSCC, both in tumor tissue and in samples from the surrounding oral mucosa, with several major periodontal pathogens commonly reported across studies, including *P. gingivalis*, *F. nucleatum* group, *Prevotella* spp. (e.g., *P. intermedia*, *P. melaninogenica*), and *Tannerella forsythia* (*T. forsythia*), as well as other periodontitis-associated anaerobes such as *Parvimonas* spp. and *Peptostreptococcus* spp. [16,17,18,19,20,21,22,23,24,25,26,27,28,29,30,31,32,33,34] (Table 1). While these findings do not provide a direct causal link, the consistent presence of certain bacteria in OSCC patients and their absence in control subjects strongly suggests they may act as contributing factors in the carcinogenic process.

#### 2.1.2. Esophageal Squamous Cell Carcinoma

Esophageal cancer is one of the most common cancers worldwide, with an incidence of approximately 450,000 cases annually [35] and a 5-year survival rate of 15–25% [36]. The majority of esophageal cancer is esophageal squamous cell carcinoma (ESCC) (88% of cases), while the remaining 12% of cases are classified as esophageal adenocarcinoma (EAC) [37]. The etiopathogenesis of esophageal cancers is multifactorial, with traditional risk factors such as chemical injury, tobacco smoking, alcohol consumption, and betel nut chewing [38], and in recent years, oral and gut microbiota dysbiosis has arisen as an additional risk factor [39].

Several case–control studies have revealed a different population of esophageal and oral microbiota in ESCC patients compared to healthy individuals by quantitative polymerase chain reaction (qPCR) or 16S rRNA sequencing [40,41,42,43,44,45,46]. Among oral pathogens, *F. nucleatum*, *P. gingivalis*, *Prevotella* spp., *T. forsythia*, *Streptococcus* spp., etc., have been linked to ESCC (Table 2). Nearly all studies report that a major finding in esophageal carcinoma is the disruption of normal oral flora, marked by a decline in commensal bacteria and a corresponding increase in pathogenic species.

#### 2.1.3. Gastric Cancer

Gastric cancer (GC) is the fifth most common cancer worldwide [54]. Well-recognized risk factors include dietary habits, alcohol consumption, tobacco smoking, and *Helicobacter pylori* (HP) infection [55,56]. Lately, microbial dysbiosis has also been recognized as an important risk factor for alimentary tract cancers [54]. In terms of the link between oral microbiota and GC, some studies showed strong risk associations between periodontal disease, tooth loss, and GC [57].

The most abundant microorganisms in GC are opportunistic pathogens or commensals of the oral cavity, such as the genera *Aggregatibacter*, *Alloprevotella*, and *Neisseria*, and the species *Streptococcus mitis*/*oralis*/*pneumoniae* [58]. Studies have shown that different species of *Streptococcus* spp. play an important role in cancer, affecting the occurrence and development of tumors through various metabolite changes and regulation of the immune microenvironment. *Streptococcus* spp. is a dominant genus in GC flora [59,60,61,62]. Other bacterial species of the oral cavity (as shown in Table 3), including *Leptotrichia* spp., *Fusobacterium* spp., *Haemophilus* spp., *Veillonella* spp., *Campylobacter* spp., etc., have higher relative abundances in patients with GC compared to controls [63]. Nevertheless, these results should be interpreted with caution, since heterogeneous sampling approaches were employed across studies, including not only gastric biopsies and gastric washings but also fecal and tongue swab specimens (Table 3).

**Table 3:** Presence of oral bacteria in various samples collected from gastric cancer patients.

Study Sample	Sample Type	Oral Bacteria and Main Findings	Ref.
GC, *n* = 57 Controls, *n* = 80	Tongue swab	The relative increase in Firmicutes and the reduced abundance of Bacteroidetes were associated with increased risk of GC; greater abundance of *Alloprevotella* spp., *Veillonella* spp., and *Streptococcus* spp. trended with higher risk of GC	[64]
GC, *n* = 12 Controls (functional dyspepsia), *n* = 20	Gastric biopsy	Several bacterial taxa were enriched in GC, such as *Veillonella* spp., *Fusobacterium* spp., *Leptotrichia* spp., and *Campylobacter* spp.	[63]
GC, *n* = 103 Controls (chronic gastritis), *n* = 212	Gastric biopsy	Proteobacteria, Firmicutes, *Fusobacterium* spp., Actinobacteria, and Nitrospirae were enriched in GC	[65]
GC, *n* = 162 Controls (non-cancerous tissue), *n* = 62	Gastric biopsy	The bacterial taxa enriched in the cancer samples were Proteobacteria, Firmicutes, *Bacteroides* spp., Actinobacteria, and *Fusobacterium* spp.	[66]
GC, *n* = 6 Gastritis, *n* = 5	Gastric wash sample	*Neisseria* spp., *Alloprevotella* spp., *Aggregatibacter* spp., and *Streptococcus* spp. were the most representative taxa abundant in GC	[62]
GC, *n* = 116 Healthy control, *n* = 88	Feces	*Veillonella* spp. and *Streptococcus* spp. were enriched in GC and showed good performance in distinguishing GC patients from healthy controls	[59]
GC, *n* = 134 Healthy control, *n* = 58	Feces	*S. mitis* and *S. salivarius* in feces were associated with a higher risk for GC; they may be associated with GC through influencing the amino acid metabolism	[60]
GC, *n* = 38 Healthy control, *n* = 35	Feces	Enterobacteria, *Streptococcus* spp., and *Escherichia* spp. were increased in the GC	[61]
GC, *n* = 22 Healthy control, *n* = 30	Feces	*Prevotella* spp. and *Streptococcus* spp. were more abundant in patients with GC	[62]

#### 2.1.4. Colorectal Cancer

Colorectal cancer (CRC) is among the three most common malignancies, and it is the second leading cause of cancer-related mortality in the world [67], comprising 11% of all cancer diagnoses [68]. It is estimated that there were 1.8 million new cases of CRC in 2018, with a much higher incidence among men than women. CRC is three to four times more common in developed than in developing nations that are adopting the modern way of life; thus, obesity, sedentary lifestyle, red meat consumption, alcohol, and tobacco are considered the driving factors behind the growth of CRC [69]. Factors affecting the incidence of CRC are age, gender, race, the human development index (HDI), body weight, diet, smoking, alcohol consumption, and chronic inflammatory bowel diseases [67,70]. There are cases of CRC that can develop from a genetic background combined with environmental factors (between 2% and 5% of all colon cancers), including familial adenomatous polyposis, Lynch syndrome, and certain hamartomatous polyposis conditions and faMUTYH-associated polyposis [70].

Even though there are numerous well-known risk factors, there are CRC cases that cannot be explained, and the need to define other causative factors arises. Gut dysbiosis (disruption of the gut microbiota) has been shown to underlie a variety of intestinal diseases, including CRC [71]. Besides this, in the past 10 years, a significant number of studies has been published detecting oral bacteria in samples (stool or tissue biopsies) taken from patients with CRC (Table 4).

Two main oral microbes associated with CRC are the *F. nucleatum* group and *P. gingivalis*. Bacteria from the *F. nucleatum* group, although common oral commensals, exhibit opportunistic pathogenic behavior in dysbiotic conditions and are among the early colonizers capable of bridging aerobic and anaerobic communities in the biofilm. The circulatory system, both blood-borne and lymphatic dissemination, seems to be the dominant way by which the *F. nucleatum* group reaches the colorectum in periodontitis patients via constant bacteremia connected with everyday habits such as chewing and tooth brushing [72]. In addition to this, oral microbial translocation through the gastrointestinal tract is also possible, as the *F. nucleatum* group has been shown to exhibit notable resistance to gastric acidity [73]. Some authors even proposed the potential of stool DNA testing for the *F. nucleatum* group by droplet digital PCR, using the presence of bacteria as a CRC tumor marker [74].

*P. gingivalis* is another pathogen that has been investigated for its cancerogenic effect in CRC [75]. The probable transmission route of *P. gingivalis* from the oral cavity to the gut is through continuous swallowing, since this bacterium is capable of resisting the acidic gastric environment [76]. Sobocki et al. showed a direct link between the abundance of *P. gingivalis* in the oral cavity and in the gastrointestinal tumors microenvironment [77].

**Table 4:** Oral microorganisms’ abundance associated with CRC.

Study Sample	Sample Type	Oral Bacteria and Main Findings	Ref.
30 CRC and 30 healthy controls	Unstimulated saliva, cancer tissues/biopsies, and stools	α and β diversity of the salivary and mucosal microbiome were higher for CRC	[78]
14 CRC patients	Biopsy and saliva samples	*F. nucleatum* group was isolated from 57.1% of CRC biopsies; an identical strain of bacteria from the *F. nucleatum* group was found in CRC and saliva in 40% of patients	[79]
101 CRC patients	CRC tissue samples and healthy tissue 10 cm beyond cancer margins	The abundance of *F. nucleatum* group bacteria in CRC tissues was significantly higher than that in normal controls	[80]
19 CRC patients	CRC tissue samples and non-neoplastic mucosa from the proximal resection margin	The abundance of *Fusobacterium* and *Campylobacter* spp. was significantly higher in the tumor	[81]
44 pieces of tissue from the tumors of 11 patients with CRC	CRC tissue	*Fusobacterium* and *Bacteroides* as the most dominant genera in the CRC	[82]
807 tumor tissues from patients with CRC	CRC tissue	17 bacterial species, including 4 *Fusobacterium* spp., classified as orally derived, were enriched in inflamed tumors	[83]
CRC (99 subjects), colorectal polyps (32), or controls (103)	Oral swabs, colonic mucosae, and stool	*Streptococcus* spp. and *Prevotella* spp. were differentially abundant in CRC compared with controls	[84]
59 patients undergoing surgery for CRC, 21 individuals with polyps, and 56 healthy controls	Fecal and mucosal samples	Increased abundance of *Bacteroides*, *Roseburia*, *Ruminococcus*, and *Oscillibacter*, among others, and genera previously reported as oral pathogens (such as *Porphyromonas*, *Peptostreptococcus*, *Parvimonas*, and *Fusobacterium*, among others)	[85]
252 CRC subjects	Fecal samples	Elevated relative abundance of members of *F. nucleatum* group, *Peptostreptococcus stomatis*, *Gemella morbillorum*, and *Parvimonas micra*	[86]

### 2.2. Other Cancer Types

#### 2.2.1. Lung Cancer

Traditionally, the lungs have been considered a sterile region of the human body [87]. In recent years, culture-independent techniques have proved that the respiratory tract, including the lungs, possesses its own microbiota [88,89,90,91]. Although this microbiota is modest (10^3^–10^5^ cells/g of tissue) [90,92] compared to microbiota of the skin or gut (10^11^–10^12^ cells/g of luminal content in gut) [93], its role in homeostasis and disease is important. After discovering the lungs’ microbiota in health, researchers focused on revealing its origin. Numerous studies have proved that lung microbiota are most similar to oral microbiota [94,95,96,97].

Research also showed that the microbiota of healthy and diseased lungs differ [95,98], and the role of dysbiosis, primarily of microorganisms originating from the oral cavity, has been considered in many lower respiratory tract diseases such as chronic obstructive pulmonary disease (COPD), asthma, pulmonary fibrosis, and even lung carcinoma [99,100,101]. Found microbiota both at health and disease differ among the studies and depend on sampling and isolation methods. The most reliable theory is that healthy lung microbiota are transient [102]. The most common isolated genera in health are *Prevotella*, *Veillonella*, and *Streptococcus* [88,94,96,103]. Some authors stated that dysbiosis of the oral microbiome may initiate oral and respiratory diseases, and vice versa, these diseases may additionally influence the progression of dysbiosis.

Analysis of the relation between the microbiome and lung cancer has just begun, and these studies are trying to explain those cases that are not connected with traditional risk factors, such as smoking, which accounts for 25–50% of cases [104]. It is noteworthy to mention that risk factors for lung cancer development—smoking and air pollution—also affect the microbiome.

Study design and sampling are heterogeneous throughout the studies. Studies can be roughly divided into those that detect microorganisms “directly” from the respiratory tract—from bronchoalveolar lavages, bronchoscopy samples, or lung tissues—and another group that detect microorganisms in saliva, buccal swabs, dental biofilm, or specific serum immunoglobulins (Ig) to some bacteria. The first group showed the presence of oral *Streptococci* [94] or *Prevotella*, *Blautia*, *Veillonella*, *Haemophilus*, *Megasphaera*, *Klebsiella*, *Rothia*, *Neisseria*, *Acinetobacter*, *Campylobacter*, *Blastomonas*, and *Porphyromonas* [5,105,106,107,108]. Some studies showed the presence of normal lung microbiota in cancer samples but with reduced diversity as a marker of cancer [109]. Another group of studies that detected microorganisms in the oral cavity showed a higher incidence of *Aggregatibacter*, *Streptococcus*, *Rothia*, *Veillonella*, *Capnocytophaga*, *Neisseria*, *F. nucleatum* group, *S. mitis*, and *Kingella denitrificans* [110,111,112], or higher levels of specific serum antibodies against antigens of *A. actinomycetemcomitans*, *P. gingivalis*, and the *F. nucleatum* group in subjects with lung cancer [113]. Further studies are required due to the fact that metabolites, toxins, and aggressive enzymes of oral bacteria can also impact lung tissue through microaspiration.

Lower alpha diversity has been shown in the saliva of subjects with lung carcinoma (smokers and non-smokers) compared to healthy controls [114,115]. In the saliva of subjects with LC, phyla Fusobacteria and Proteobacteria, family Actinomycetaceae, genera *Fusobacterium*, *Neisseria*, and *Capnocytophaga*, and species *Kingella denitrificans* and *S. mitis* were upregulated.

An important study by Liu et al. showed a higher presence of *P. gingivalis* in carcinoma tissue than in carcinoma-adjacent tissue. This study also marked *P. gingivalis* as a prognostic marker of 5-year survival [108].

#### 2.2.2. Breast Cancer

Breast cancer is one of the most frequent cancers worldwide, with a 5-year worldwide prevalence of more than 8,000,000 [116]. Female breast cancer (FBC) is about 70–100 times more frequent than male breast cancer (MBC) [117]. Despite well-known intrinsic (age, gender, race, family susceptibility, natural hormonal changes, and proliferative benign lesions of the mammary gland) and extrinsic (dietary habits, obesity, and hormonal therapy) risk factors associated with breast cancer development [118], many cases are not related to any of them. Likewise, for many other cancers, these cases may be related to the microbiome to some extent. Similarly to lung tissue, the breast has been traditionally considered sterile tissue, but the presence of breast microbiota has been proven [119,120]. Some studies even speculate that breast tissue may have its own microbiota, which differs from other body sites, and the frequent presence and high abundance of specific microorganisms (Proteobacteria and Firmicutes, specifically class Bacilli) are a consequence of adaptation of these microorganisms to the fatty acid microenvironment of breast tissue [119]. Other genera present in normal healthy breast tissue were *Acetobacter* [121], *Stenotrophomonas*, *Caulobacter*, *Vibrionimonas*, *Amphibacillus* [122], and *Xanthomonas* sp. [121].

The first studies that connected the microbiome and breast cancer date back to the 1990s [123,124]. The studies are heterogeneous both in sampling, microbial detection procedures, and the potential role of the microbiome in cancerogenesis. In this review, we will focus only on studies that used breast tissue as a sample. Some studies indirectly showed a relationship between breast cancer and microorganisms, through decreased survival rates of BC patients with antimicrobial exposure of BC patients [125].

Some studies showed differences in the breast tissue of benign lesions or high-risk healthy tissues compared to healthy tissue. For example, Tzeng et al. showed a higher mean relative abundance of *Propionibacterium*, *Finegoldia*, *Granulicatella*, *Streptococcus*, *Anaerococcus*, *Ruminococcaceae* UCG-002, *Corynebacterium* 1, *Alicyclobacillus*, *Odoribacter*, and *Escherichia/Shigella* [122] in benign lesions compared to healthy tissue. Additionally, some articles showed that the microbiome of healthy control tissue is different from adjacent non-tumorous tissue [121,122], and that the microbiome of adjacent non-tumorous tissue is similar to the tumor tissue microbiome [121].

Microbiota have also been connected with the tumor grade or type. Higher stage tumors were connected with a higher abundance of *Porphyromonas*, *Lacibacter*, *Ezakiella*, and *Fusobacterium* [122].

Also, it has been shown that different types of tumors are related to different microbiomes. *Tepidiphilus*, *Alkanindiges*, and *Stenotrophomonas* were dominant in invasive ductal carcinoma (IDC), while *Peptostreptococcus*, *Micromonospora*, *Faecalibacterium*, and *Stenotrophomonas* were dominant in invasive lobular carcinoma (ILC) [122]. Banerjee et al. showed a difference in microbiological signature between four types of breast cancer: estrogen receptor-positive (ER+), human epidermal growth factor receptor 2-positive cancer (HER+), triple-receptor-positive cancer (positive for ER, HER2, and progesterone receptor), and triple-negative cancer [126]. Although every type of tumor showed its specific microbiota, the genera *Actinomyces*, *Bartonella*, *Brevundimonas*, *Coxiella*, *Mobiluncus*, *Mycobacterium*, *Rickettsia*, and *Sphingomonas* were associated with all tumor types [126].

Although male breast cancer is rare, Niccolai et al. showed that there is a difference between the microbiota composition of male breast cancer and female (both in cancer tissue samples and adjacent tissue samples) [127]. In adjacent tissues, male samples showed greater diversity than female samples. The differences between genders were less expressed in cancer tissue than in adjacent tissue. Male cancer tissue showed higher abundance of some orders (Burkholderiales, Caulobacterales, and Pseudomonadales), families (Comamonadaceae), and genera (*Actinomyces* spp., *Mycoplasma* spp.) than female cancer tissue. On the other hand, female breast cancer tissue showed a higher abundance of Clostridiales, *Actinomyces* spp., *Halomonas* spp., *Prevotella* spp., and Streptococcaceae [127]. Diversity between the tumor and adjacent tissue was assessed for male cancer tissue. Some studies did not find a difference in diversity between tumorous and adjacent tissues in female subjects, which can lead to the conclusion that female subjects are prone to cancerogenesis throughout the whole breast tissue [127,128].

Besides that, the microbiomes of cancer and healthy tissue are different. Hieken et al. showed that the microbiota of healthy tissue adjacent to invasive cancer are different from healthy tissue adjacent to benign lesions [129].

#### 2.2.3. Pancreatic Cancer

Pancreatic cancer (PC) is currently the fourth leading cause of cancer-related death worldwide. The prognosis of patients with PC is generally poor, with a 5-year overall survival rate of 9% [130]. Pancreatic ductal adenocarcinoma (PDAC) is among the most aggressive and least treatable forms of cancer [131]. Known risk factors for PC include tobacco smoking, obesity, type II diabetes, and chronic pancreatitis. [132,133,134]. Recent clinical and preclinical studies highlight the emerging roles of the microbiota in patients with PCs. Several prospective studies show that oral microorganisms and periodontal diseases are associated with a high risk of pancreatic cancer [135,136,137].

The study by Farrell et al. was the first study that predominantly measured variations in salivary microbiota and evaluated its association with pancreatic cancer. *Neisseria elongata* and *S. mitis* were decreased in PC compared with healthy controls. *Veillonella*, *Campylobacter*, and *Prevotella* were the most dominant oral microorganisms in the saliva of patients with cancer [138].

Later studies also showed differences in microbial composition of the microbiota in the saliva of patients with PC, showing higher levels of *Leptotrichia*, *P. gingivalis*, *A*. *actinomycetemcomitans*, and *Streptococcus* spp. than in the saliva of healthy control subjects [139,140]. Also, higher levels of *Leptotrichia* sp. and *Fusobacterium* sp. were found on the tongue of patients with PC compared to the healthy controls [141].

Michaud et al. evaluated pre-diagnostic blood samples from patients who subsequently developed PC compared to matched healthy controls. Antibodies against a preselected panel of known oral bacteria were measured, and it was shown that individuals with high levels of antibodies against *P. gingivalis* had a higher risk of developing PC [142].

A study by Del Castillo et al. analyzed tissue samples obtained from 50 subjects with pancreatic cancer, and the relative abundance of *Fusobacterium* spp. was higher in cancer subjects compared with non-cancer subjects [143].

#### 2.2.4. Urogenital Cancers

Emerging evidence suggests a bidirectional link between oral health and urogenital cancers. Researchers have detected oral microbes in the female genital tract and urinary system, indicating the presence of an “oral-genitourinary axis”. In other words, bacteria commonly found in the mouth can colonize urogenital sites [144]. Potential routes for the translocation of periodontal pathogens include hematogenous spread (bacteria entering the bloodstream through inflamed gums) or direct transmission via oral–genital contact (e.g., oral sex) [145]. This dynamic exchange means that a dysbiotic oral microbiome (observed in periodontitis) may seed or influence the microbial communities in the urogenital tract, potentially providing a suitable niche for malignancy.

An increased quantity of *Prevotella* spp. was found in the urine and tumor tissue of bladder cancer patients compared to healthy controls [146]. Certain *Prevotella* were also abundant in the vaginal microbiomes of cervical cancer patients [147].

Epidemiological data show that a weak immune response against *T. forsythia* correlates with higher bladder cancer incidence, thus suggesting *Tannerella* infection to be a silent contributor to bladder carcinogenesis [148].

*P. gingivalis* DNA has been found in some urogenital tumor samples, and its presence is linked to elevated local inflammation [145].

As oral microbes with demonstrated tissue-invasive capacity, the *F. nucleatum* group has been detected in urogenital tumors, where it forms biofilms and modulates the local immune microenvironment. These findings are in line with its classification as a “mobile microbiota” member capable of translocating from the mouth to other mucosal sites, including the cervicovaginal and urinary tracts [145,149].

#### 2.2.5. Liver Cancer

Recent studies have ignited interest in understanding the potential connection between oral microorganisms and liver cancer, particularly hepatocellular carcinoma (HCC). The liver, a vital organ with a central role in metabolism, is susceptible to various risk factors, including chronic inflammation. Emerging evidence suggests that oral bacteria may contribute to liver carcinogenesis. Oral bacteria, as well as their metabolites, which can translocate from the oral cavity to the gut, can influence the gut microbiota and subsequently impact liver function. Moreover, the role of inflammation, often associated with periodontal diseases, is implicated in the promotion of liver cancer. Various studies show that the oral microbiome differs in patients with HCC compared to healthy individuals [150,151,152,153,154].

This microbial dysbiosis in liver cancer patients shows higher levels of *Fusobacterium*, *Leptotrichia*, *Actinomyces*, and *Campylobacter* compared to the levels in healthy individuals [150]. Another study of the oral microbiome in liver cancer patients showed a high abundance of *Haemophilus* and *Porphyromonas*, while the levels of bacteria from the genera *Moryella*, *Leptotrichia*, *Dialister*, *Serratia*, *Enterococcus*, and *Actinobacillus* were lower in samples from patients compared to controls without liver cancer [152].

A study on non-alcoholic steatohepatitis (NASH)-related HCC patients also showed elevated levels of *P. gingivalis*, along with members of the *F. nucleatum* group in the saliva. Along with this finding, the serum levels of Ig G antibody against *P. gingivalis* and the *F. nucleatum* group were significantly higher in NASH-HCC patients than in the NASH patients without HCC [151].

## 3. Pathogenic Mechanisms

The pathogenic contribution of oral bacteria to carcinogenesis involves multiple biologically distinct yet interconnected processes. These include persistent inflammatory responses, direct genotoxic effects, the modulation of host immune surveillance, and activation of oncogenic signaling pathways, collectively facilitating tumor initiation, progression, and metastatic potential.

### 3.1. Chronic Inflammation

**Figure 1:**
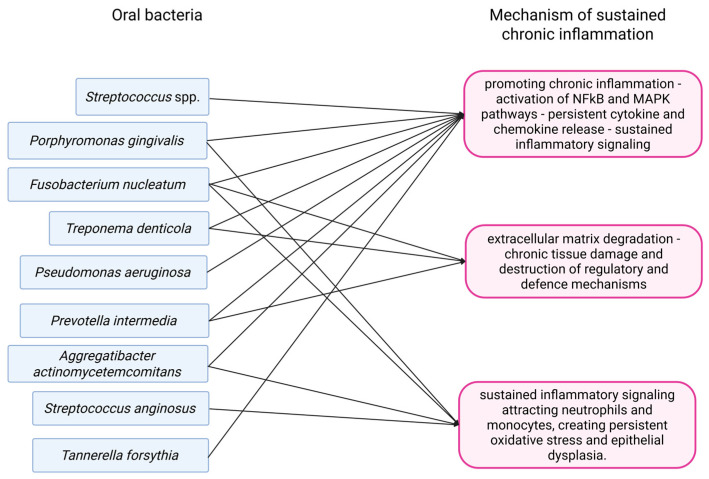
A summation of mechanisms through which oral bacteria cause chronic inflammation.

An overview of mechanisms by which oral bacteria lead to sustained chronic inflammation is given in Figure 1.

#### 3.1.1. Oral Squamous Cell Carcinoma

Several inflammatory cytokines and chemokines from the CXC family have been shown to enhance the development of OSCC through promoting cell migration and proliferation [155]. *P. gingivalis* induces the secretion of many cancer-contributing chemokines and cytokines, including Interleukin (IL)-1β, IL-6, IL-8, transforming growth factor (TGF)-β1, epidermal growth factor (EGF), and tumor necrosis factor alpha (TNF-α) [156,157]. New et al. discovered that the release of tumor-promoting substances, including IL-6 and IL-8 by cancer-associated fibroblasts (CAFs), is associated with HNSCC and plays a role in its development [158].

Gram-negative bacteria endotoxins (LPSs) induce the release of TNF-α, which exerts its effect through the nuclear factor kappa B (NF-κB) pathway, known to be upregulated in OSCC [159,160]. The *F. nucleatum* group in human epithelial cells enhances cellular migration by potentially activating Etk/BMX, S6 kinase p70, and RhoA kinase and increasing the synthesis of matrix metalloproteinase (MMP)-13 (collagenase 3) via activation of the mitogen-activated protein kinase p38 [161].

Another periopathogen, *Treponema denticola* (*T. denticola*), is characterized by its strong proteolytic activity, mostly attributed to the secretion of dentilisin, a chymotrypsin-like proteinase (Td-CTLP). Dentilisin can break down IL-8 and TNF-α, as shown in studies by Jo et al. [162], and consequently modulates the innate immune response. Additionally, it can convert pro-MMP8 and pro-MMP9 into their active forms, degrading the proteinase inhibitors TIMP-1, TIMP-2, and α-1-antichymotrypsin, as well as complement C1q [163].

*P. aeruginosa* can promote inflammation via ExoU by triggering the NF-kB pathway, resulting in the release of IL-8 [164]. A significant increase in MMP9 gene expression was also observed in *P. intermedia* OSCC-positive cases [165]. *A. actinomycetemcomitans* and the members of the *F. nucleatum* group can upregulate the production of CCL20 in oral cancer cell lines and induce the release of the proinflammatory cytokines [166,167,168,169].

#### 3.1.2. Esophageal Squamous Cell Carcinoma

Based on the close anatomical proximity between the oral cavity and esophagus, *P. gingivalis* in the esophagus also causes chronic inflammation of the normal esophageal mucosa and may promote tumor progression and chemotherapy resistance [170].

The researchers also found that *T. forsythia* was associated with a higher risk of EAC, whereas *P. gingivalis* was associated with a higher risk of ESCC [49]. *T. forsythia* is known to be a periodontal pathogen. Its virulence is enabled thanks to the O-glycan structures, present in the S-layer of this bacterium, which likely play a crucial role in the development of infection [171].

The literature reports indicate a positive correlation between cyclooxygenase 2 (COX-2) expression and the development of tumors and metastatic sites. Activation of COX-2 is influenced by proinflammatory cytokines and stress factors. Expression of the COX-2 gene is stimulated by factors involved in the inflammatory reaction, such as IL-1 and TNF-α, both of which can be generated by *T. forsythia*, LPSs, transcription factors, and oncogenes [172].

The *F. nucleatum* group can stimulate the secretion of MMP-9 and MMP-13 from epithelial cells, leading to the degradation of collagen IV in the basement membrane and extracellular matrix which facilitates tumor progression, including invasion, metastasis, growth, and angiogenesis in the esophagus [173].

Narikiyo et al. [174] demonstrated that *S. anginosus* causes the attraction and activation of neutrophils and monocytes by releasing chemokines, thereby leading to the development of epithelial dysplasia and potentially cancer. From their findings, the frequent presence of *T. denticola* in esophageal cancers supports a model in which this pathogen contributes to carcinogenesis through persistent mucosal colonization that drives chronic low-grade inflammation, NF-κB-mediated cytokine signaling, and proteolytic tissue disruption, creating a microenvironment conducive to malignant transformation [174].

#### 3.1.3. Lung Cancer

The most commonly mentioned mechanism of lung cancer etiopathogenesis that can be connected with dysbiosis is inflammation [175], driven by *P. gingivalis* and *A. actinomycetemcomitans*.

The first step in recognition of both normal microbiota and pathogens is the activation of toll-like receptors (TLRs), which directly recognize the pathogen-associated molecular patterns (PAMPs) of microorganisms and thereby determine immune tolerance or response [176]. Additionally, these receptors can recognize damage-associated molecular patterns (DAMPs) released from the damaged tissues. Ten TLRs can be found in various human cells, including some lung cancer cells [177]. Generally, the induction of inflammatory cytokines such as TNF-α, IL-1β, IL-6, and type I interferons is the final outcome of TLR activation, although via different intracellular mechanisms.

*A. actinomycetemcomitans* produces cytolethal distending toxin (CDT), which can play a role in tumorigenesis by inducing chronic inflammation through stimulating interferon (IFN)-γ, IL-1β, and IL-6 [178,179]. Besides the role of CDT, LPS also induces inflammatory mediators such as IL-1β, IL-6, IL-8, and TNF-α.

#### 3.1.4. Colorectal Cancer

The most important virulence factor of *P. gingivalis* is the endotoxin LPS, which induces systemic inflammation through increased release of proinflammatory mediators as part of the host’s immune response to LPS [180].

*P. gingivalis* promotes tumorigenesis by recruiting tumor-infiltrating myeloid cells and creating a proinflammatory microenvironment via activation of the hematopoietic NOD-like receptor protein 3 (NLRP3) inflammasome [181].

Gingipains and other virulence factors of *P. gingivalis* modulate cellular homeostasis and increase markers of both local and systemic inflammation [182].

Tumor-associated macrophages (M2-type macrophages) are derived from precursor myeloid-derived suppressor cells (MDSCs), which are involved in immune suppression, thus promoting angiogenesis and carcinogenesis [183,184]. In human CRC, *F. nucleatum* group infection promotes M2-type macrophage polarization and tumor growth and progression in a TLR4-dependent manner by activating the IL-6/p-STAT3/c-MYC and the TLR4/NF-ĸB/S100A9 signaling pathways [185,186,187].

#### 3.1.5. Pancreatic Cancer

Tan et al. showed that *P. gingivalis* promoted PC progression through the secretion of neutrophilic chemokines and neutrophil elastase [188].

Udayasuryan et al. showed that *F. nucleatum* group infection in the pancreas elicits cytokine secretion from cancer cells and promotes tumor-associated phenotypes in PDAC cells that are associated with tumor progression [189]. Hayashi et al. compared clinical features with colonization by members of the *F. nucleatum* group in pancreatic cancer tissues. Their findings indicate that interactions between cancer cells and intratumor bacteria can affect the progression of pancreatic cancer. They demonstrated that the *F. nucleatum* group promoted the C-X-C motif chemokine ligand 1 (CXCL1) secretion from pancreatic cancer cells, leading to cancer progression through autocrine signaling [190].

During infection and invasion by members of the *F. nucleatum* group, after binding with the host cell toll-like receptor 4 (TLR4), the produced LPS interacts with the Toll/il-1 receptor (TIR) domain-containing adaptor, triggering IFN-β (via TRIF) and subsequently myeloid differentiation primary response protein 88 (MyD88) recruitment. MyD88 induces IRAK (IL–1 receptor-associated kinase) phosphorylation, which dissociates from the receptor, interacts with adaptor proteins TNFR-associated factor 6 (TRAF6) and TAK1-binding protein 2 (TAB2) on the membrane, and regulates their transport to the cytosol. Subsequently, TRAF6 becomes ubiquitinated (Ub) and activates TAK1 (TGF-β-activated kinase 1), which phosphorylates and activates the IκB kinase (IKK) complex. IKK phosphorylates IκB, an inhibitor of NF-κB, thereby allowing NF-κB to be rapidly activated and translocated to the nucleus, promoting the expression of related genes by binding to κB [191,192].

NF-κB is a multifunctional dimeric transcription factor that coordinates cell proliferation and is closely related to cancer development. It has been reported that high levels of MyD88 promote PDAC cell growth and are associated with poor survival in patients with PDAC [193].

#### 3.1.6. Gastric Cancer

Studies have found that the gastric microbiota of patients with gastric cancer is imbalanced, and *Streptococcus* spp. are enriched in gastric cancer tissues, which are significantly different from the microbiota of healthy people or patients with chronic gastritis. *Streptococcus* spp. can produce urease, which is the main inducer of the innate immune response and is involved in the occurrence of gastric cancer [194].

#### 3.1.7. Urogenital Cancers

Oral anaerobes like *P. gingivalis* and *Prevotella* spp. stimulate the production of cytokines such as IL-6, IL-8, IL-1β, and TNF-α in affected tissues [145]. This persistent inflammatory milieu can cause DNA damage and support continuous cell proliferation. Over time, such inflammation contributes to the initiation and progression of cancer in organs such as the bladder and cervix.

### 3.2. Direct Genotoxicity

**Figure 2:**
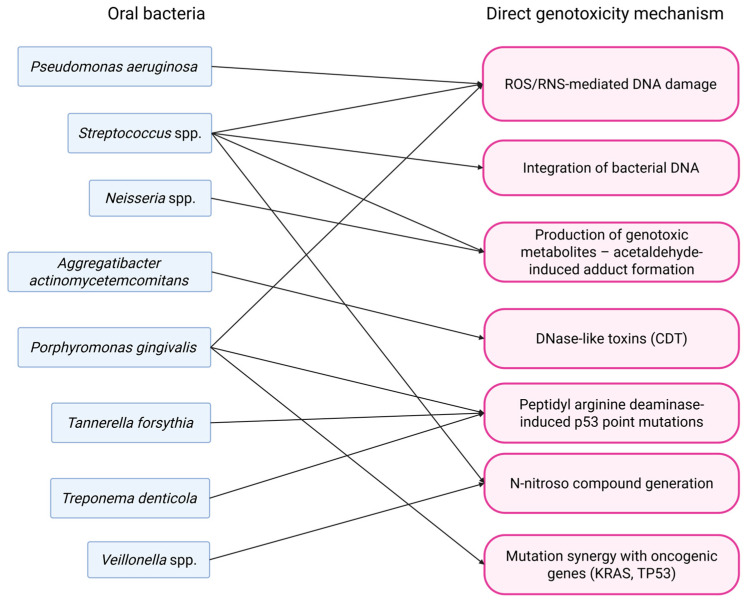
Mechanisms of oral bacteria-driven direct genotoxicity.

A summary of mechanisms by which oral bacteria lead to genomic instability via direct genotoxicity is illustrated in Figure 2.

#### 3.2.1. Oral Squamous Cell Carcinoma

*P. aeruginosa*, a pathogen that can be isolated from periodontal pockets, is found in association with the *F. nucleatum* group in OSCC samples and is implicated in carcinogenesis by damaging DNA in the epithelial cells [30,195]. The presence of *S. anginosus* increases the synthesis of nitric oxide (NO) and cyclooxygenase-2 (COX-2), leading to DNA damage and the development of cancer in the infected tissue [17]. Moreover, *S. anginosus* DNA can be incorporated into the host genome, thereby causing damage [196].

Members of the genus *Neisseria*, including the species *N. mucosa*, *N. flavescens*, and *N. flava*, have the substantial ability to convert ethanol into acetaldehyde, a substance that may induce point mutations and impair DNA repair enzymes [197]. Besides these *Neisseria* strains, other studies have identified *S. salivarus*, *S. mitis*, and *Neisseria sicca* as significant acetaldehyde producers [198].

#### 3.2.2. Lung Cancer

Lung cancers have also been connected to specific mutations. The mutation of the tumor suppressor gene *TP53* is one of the most commonly seen in lung cancer [199], both in small cell and non-small cell lung cancers. p53 has a central role in response to DNA damage, thus protecting against tumorogenesis. This mutation can be caused by many endogenous and exogenous factors, and it has been proven that this mutation is related to smoking and to alterations in the lung microbiota, such as the enrichment of *Acidovorax* [200]. Observing the studies about cancer of different organs (pancreatic cancer), it has been suggested that some periodontal pathogens, such as *P. gingivalis*, *T. forsythia*, and *T. denticola*, possess the enzyme peptidyl arginine deaminase (PAD), which may cause *TP53* point mutations [201].

As mentioned previously, *A. actinomycetemcomitans* produces cytolethal distending toxin (CDT), which can also take a role in tumorigenesis via increasing genomic instability, disturbing the cell cycle [178,179].

#### 3.2.3. Gastric Cancer

Metabolic enzymes associated with denitrification, including nitrous oxide reductase and nitrate reductase, were enriched in the cancer subjects’ gastric microbiota compared to the non-cancer group [202]. *Veillonella*, as a nitrate-reducing bacterium, catalyzes nitrite production from nitrate reduction. This bacterium could be responsible for the accumulation of nitrite in the stomach, which is a precursor of endogenous N-nitroso compounds (NOCs). As NOCs have a crucial role in the development of gastric cancer, *Veillonella* may affect the carcinogenesis process through its nitrate-reducing function [203].

*Streptococcus* is also involved in the formation of NOCs [204,205], which are known to cause DNA mutations, leading to the malignant transformation of cells.

#### 3.2.4. Pancreatic Cancer

*P. gingivalis* can increase tumorigenic behavior through synergy with other oncogenic factors, such as mutant KRAS. It induces miRNA expression and may contribute to mutations in *KRAS* and *TP53*. Mutations in *KRAS* influence gut and pancreatic microbiota composition and diversity, and certain host genetic variations can cause dysbiosis and thereby lead to cancer development [206].

### 3.3. Modulation of Immune Response

**Figure 3:**
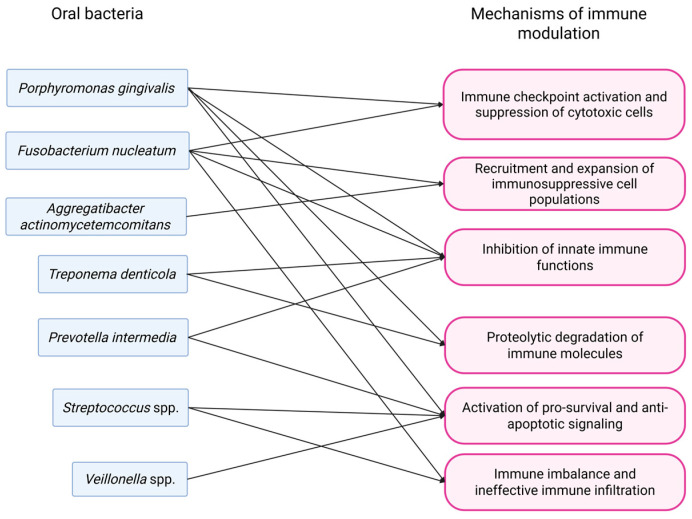
Mechanisms by which oral bacteria lead to modulation of immune response.

A summary of the mechanisms of modulation of the immune response driven by oral bacteria is given in Figure 3.

#### 3.3.1. Oral Squamous Cell Carcinoma

*P. gingivalis* can stimulate the production of programmed death ligand 1 (PD-L1, B7-H1) and B7-DC receptors in squamous carcinoma cells. These receptors cause the inactivation and death of activated T lymphocytes, allowing tumor cells to evade the host immune response [207]. Liu et al. [208] observed the ability of *P. gingivalis* to hinder the phagocytosis of Cal-27 OSCC cells by macrophages, suggesting its role in immunoevasion. The levels of the *F. nucleatum* group in OSCC showed a substantial negative correlation with markers of B-lymphocytes, CD4+T-helper lymphocytes, M2-macrophages, and fibroblasts, which may suggest their contribution to the host’s immune response against tumors [209]. The *F. nucleatum* group can shield tumors from immune cell assault by stimulating the Fap2-dependent inhibitory immunoreceptor T cell Ig, ITIM domain (TIGIT), and carcinoembryonic antigen cell adhesion molecule 1 (CEACAM1), thereby suppressing the functions of T and natural killer cells [210,211]. In OSCC, the expansion of MDSCs impair T-cell function and promote Th17 differentiation, thereby creating an immunosuppressive tumor microenvironment that supports oral carcinogenesis [212]. The members of the *F. nucleatum* group can induce the generation and recruitment of MDSCs via chemokine signaling (such as CXCL1) and NLRP3 activation, thus contributing to OSCC progression [213].

The *F. nucleatum* group produces a specific protein FAD-I (Fusobacterium-associated defensin inducer) that activates human beta defensin 2 (hBD-2) expression via TLR-1/2 and TLR-2/6 heterodimerization [214]. On the other hand, *T. denticola* possesses the capability to inhibit the production of hBD-2 and chemokine IL-8 in gingival epithelial cells, consequently also modifying the host innate immune response [215].

Increased host cell infiltration was observed as a result of the *P. intermedia* infection, as well as neutrophil disablement [216].

#### 3.3.2. Esophageal Squamous Cell Carcinoma

Median serum levels of IgA and IgG against *P. gingivalis* were significantly higher in ESCC. High serum levels of IgA or IgG against *P. gingivalis* were associated with a worse prognosis in ESCC patients [217].

The *F. nucleatum* group induces immunosuppressive myeloid-derived suppressor cell (MDSC) enrichment via activation of the NOD-like receptor protein 3 (NLRP3) inflammasome [218].

#### 3.3.3. Colorectal Carcinoma

*P. gingivalis* can produce gingipains, a family of cysteine proteases that can degrade extracellular matrix components, such as Igs, cytokines, complement, and collagen, enabling bacteria to evade host reaction, promote pathogenic microbiome expansion, and contribute to carcinogenesis [219].

High amounts of intratumoral bacteria of the *F. nucleatum* group are associated with a high density of CD68+ tumor-infiltrating macrophages in the microsatellite instability-high (MSI-H) molecular subtype of CRC (MSI-H CRCs) [220]. In an animal model study, mice were fed with the *F. nucleatum* group, which induced the proliferation of CD103+ dendritic cells (DCs) and the subsequent expansion of Foxp3+ regulatory T cells, a CD4+ T-cell subset that effectively inhibits cytotoxic and effector T cells inside the tumor tissue [221,222].

The *F. nucleatum* group is capable of reducing NK cell activity if there is sufficient bacterial load in the gut. The bacterial membrane protein Fap2 can mediate bacterial enrichment in CRC by binding to tumor-expressed Gal-GalNAc [223]. Suppression of the immune attack through the binding of bacterial Fap2 to the inhibitory immune receptor TIGIT on NK and T cells occurs after adhesin FadA promotes E-cadherin/β-catenin signaling. This activity leads to the inhibition of NK cells against tumor cells and subsequently promotes the growth and progression of CRC [224].

*F. nucelatum* group binding to host cells through Fap2 protein induces the secretion of proinflammatory cytokines IL-8 and CXCL1, which accelerates CRC cell migration [225].

#### 3.3.4. Lung Cancer

A study by Jiang et al. showed that an increase in the *F. nucleatum* group in the unstimulated saliva of lung cancer patients was associated with the downregulation of NK-cell-mediated cytotoxicity [115].

Apoptosis is an important mechanism of tumor suppression, and apoptosis evasion is one of the hallmarks of all cancer types. In cancers, the intrinsic pathway of apoptosis is usually inhibited. Some oral bacteria have been connected with anti-apoptotic activity. For example, *P. gingivalis* induces anti-apoptotic JAK1/AKT/STAT3 signaling [226], as well as the inhibition of pro-apoptotic Bad through its phosphorylation [227].

Tsay et al. [106] showed that PI3K signaling pathways and ERK (extracellular signal-regulated kinases) were upregulated in lung cancer patients, together with the enrichment of *Streptococcus* and *Veillonella*. This study additionally showed that in vitro exposure of A549 cells (airway epithelial cells) to *Veillonella*, *Streptococcus*, and *Prevotella* upregulated PI3K signaling pathways and ERK. These pathways, consisting of kinase cascades, regulate cell proliferation, differentiation, and survival [178]. More importantly, activation of PI3K is an early event in lung cancer etiopathogenesis [179].

#### 3.3.5. Gastric Cancer

The abundance of *Streptococcus* was positively correlated with the number of CD3+ T cells and negatively correlated with the number of NK cells [228].

#### 3.3.6. Pancreatic Cancer

*P. gingivalis* LPS upregulated the expression of regenerating islet-derived 3G (Reg3G) in pancreatic tissue. The overexpression of Reg3G has been found to accelerate tumor growth and promote an immunosuppressive microenvironment [229]. Gnanasekaran et al. [230] demonstrated that *P. gingivalis* directly affects PDAC cells, inducing cell proliferation, which is enhanced in hypoxic conditions characteristic of pancreatic carcinoma. This promotion of proliferation is linked to the intracellular survival of the bacteria and its ability to augment AKT signaling and cyclin D1 expression, two of the crucial pathways implicated in PDAC progression.

Intratumor presence of the *F. nucleatum* group suppressed tumor-infiltrating CD8+ T cells by recruiting myeloid-derived suppressor cells (MDSCs) [190].

#### 3.3.7. Urogenital Cancers

*P. gingivalis*, for example, produces proteases (gingipains) that cleave immune signaling molecules and can activate anti-apoptotic pathways (JAK/STAT, etc.), thereby helping infected cells survive [145]. It also secretes enzymes, such as peptidyl-arginine deiminase (PAD), that can inhibit tumor suppressor proteins, for instance, by contributing to *TP53* mutations [144]. The *F. nucleatum* group can bind to inhibitory receptors such as TIGIT on natural killer (NK) cells via its Fap2 protein, which protects tumor cells from immune attack [145]. The net effect is an immunosuppressive tumor microenvironment in which cancer cells escape immune surveillance.

### 3.4. Epigenetic Alterations and Oncogenic Signaling Pathways Activation/Modulation

**Figure 4:**
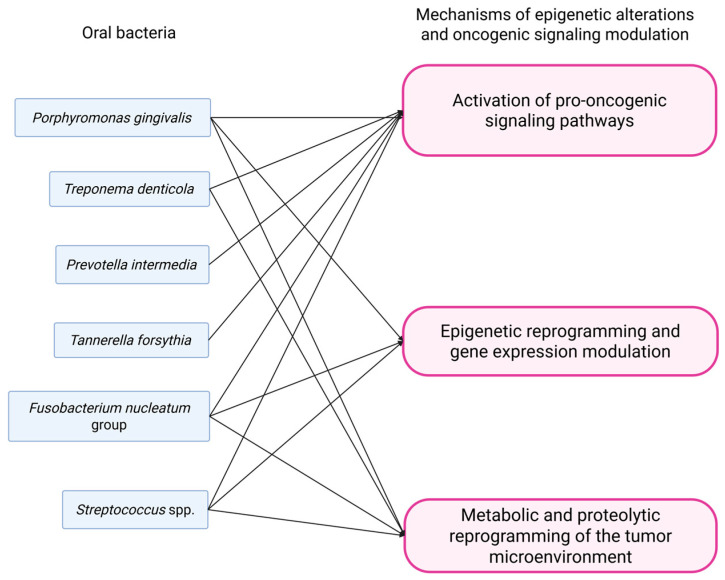
Bacteria-driven mechanisms of epigenetic alterations and oncogenic signaling modulation.

An overview of mechanisms of epigenetic alterations and oncogenic signaling modulation is presented in Figure 4.

#### 3.4.1. Oral Squamous Cell Carcinoma

OSCC cells exhibited the upregulated expression of NF-κB and MAPK pathway genes, including IKBKB, MAPK14, MAPK8, and JUN, during infection with *P. gingivalis*, hence promoting cancer proliferation [231]. *P. gingivalis* regulated cyclin D1 expression through the miR-21/PDCD4/AP-1 negative feedback signaling pathway [232].

*P. gingivalis*—FimA fimbriae primarily promote the formation of OSCC by targeting the chemokine receptor type 4 (CXCR4) and activating the phospho-Akt1 (pAKT1)-pFOXO1-dependent pathway [233]. *P. gingivalis* can also induce the dephosphorylation and activation of FOXO1, a forkhead transcription factor involved in oxidative stress responses, inflammatory cytokine production, and cell survival [234].

The study by Woo et al. indicates that tumor xenografts consisting of OSCC cells infected with *P. gingivalis* showed increased resistance to Taxol due to the activation of Notch1 [235].

*T. denticola* also stimulates cancer cell migration and tumosphere formation, enhancing toll-like receptors (TLR/MyD88) and integrin/FAK crosstalk and signaling pathways [236]. Peng et al. [237] concluded that *T. denticola* can infiltrate Cal-27 cells and actively stimulate cell growth, control the cell cycle, prevent cell death, and increase the expression of Ki-67 by activating the TGF-β pathway. Td-CTLP was also found to have a strong correlation with TLR7, TLR9, and cytoplasmic c-Myc together with the early OSCC recurrence in younger patients [238]. *T. denticola* can produce hydrogen sulfide, which was shown to enhance the proliferation of oral squamous carcinoma cells by activating the COX2/AKT/ERK1/2 pathway [239,240].

*P. intermedia*, a member of the orange-complex bacteria, was also detected at significantly elevated levels in OSCC samples [34] and was shown to activate tyrosine kinase receptors that regulate cell growth, migration, and various differentiation pathways that are linked to disease development [241,242].

#### 3.4.2. Esophageal Squamous Cell Carcinoma

*P. gingivalis* can promote immortalized oral epithelial cell proliferation, migration, and invasion by activating ERK1/2-Ets1 and proteinase-activated receptor 2 (PAR2)/NF-κB pathways [243].

In a cell-line study, Meng et al. demonstrated that *P. gingivalis* promoted the proliferation and motility of ESCC cells by activating the NF-κB signaling pathway [244].

In an animal study, Chen et al. showed that *P. gingivalis* infection was associated with advanced stages and a poor prognosis in a carcinogen-induced mouse esophageal cancer model through the IL-6/STAT3 pathway [245].

The activation of MMPs, driven by the interaction between *P. gingivalis* fimbriae and host cells, represents another potential mechanism contributing to esophageal cancer development [246].

Overexpression of the *COX-2* gene occurs in many cancers, including esophageal cancer. The Notch signaling pathway, through which signal transduction occurs, plays a role in cell differentiation and fate determination. Notch receptors on cells interact with transmembrane ligands on adjacent cells. Inflammatory cytokines produced during the inflammation induced by *P. gingivalis* and *T. forsythia* may affect the regulation of the Notch pathway [172].

In a cell-line study, Nomoto et al. demonstrated that the *F. nucleatum* group promotes ESCC cell growth and migration by activating the NOD1/RIPK2/NF-κB pathway [247].

The *F. nucleatum* group can also induce chemoresistance in ESCC cells by modulating autophagy [248].

#### 3.4.3. Colorectal Cancer

*P. gingivalis* has two different types of fimbriae, minor fimbriae and major fimbriae, which allow bacteria to bind to host cells and invade them, causing an inflammatory reaction [249]. *P. gingivalis* can provoke cellular senescence via butyrate secretion and accelerate the onset of CRC [250].

*P. gingivalis* maintains the anti-apoptotic ability of epithelial cells through different mechanisms, including upregulation of both the PI3K/AKT [251] and JAK/STAT3 signaling pathways and inhibition of caspase-3 [201] and caspase-9 [252]. *P. gingivalis* inhibits the suppressor of cytokine signaling 3 (SOCS3) by regulating mir-203 [253], while SOCS3 can induce apoptosis via STAT3 [254].

The nucleoside diphosphate kinase secreted by *P. gingivalis* can scavenge ATP to inhibit P2X7-mediated apoptosis and promote tumorigenesis [255]. It also contributes to epithelial cell proliferation through regulating the activity of PI3K, p53 [256], and cyclins [257], as well as activation of the WNT/β-catenin pathway.

*P. gingivalis* can also induce COX-2 expression and the production of PGE2 through its influence on human monocytes (i.e., activation of both MEK/ERK/AP-1 and IkB kinase/NF-κB p65 cascades) [258]. Gingipains produced by *P. gingivalis* are essential for stimulation of the MAPK/ERK signaling pathway and for inducing CRC cell proliferation. The bacteria can adhere to CRC cells and invade them within only a few hours after administration [259].

The *F. nucleatum* group can activate lncRNA ENO1-IT1 transcription via upregulating the binding efficiency of transcription factor SP1 to the promoter region of ENO1-IT1. Elevated ENO1-IT acts as a guider module for histone acetyltransferase KAT7, specifying the histone-modification pattern on its target genes, including ENO1, and consequently altering the CRC biological function [227]. High amounts of the intratumoral *F. nucleatum* group promote CpG island hypermethylation of the *CDKN2A* (*p16*) gene in the microsatellite instability-high (MSI-H) molecular subtype of CRCs (MSI-H CRCs) [220].

#### 3.4.4. Lung Cancer

TLR4, TLR5, TLR7, and TLR8 were more highly expressed in non-small cell lung cancer (NSCLC) [260,261]. Additionally, the expression of some of these receptors was correlated with specific biological characteristics and with response to therapy [260,261]. Higher expression of these receptors in cancer may be explained by epigenetic changes, such as DNA methylation, which can be caused by oral pathogens such as *P. gingivalis* [262]. *P. gingivalis*, a major periodontal pathogen, is linked to the overexpression of TLRs in many other tissues [263,264].

Similarly to CRC [224], in lung cancer metastasis, the *F. nucleatum* group acts via FadA to invade cells and interact with E-cadherin, thereby activating β-catenin signaling pathways [265].

#### 3.4.5. Gastric Cancer

Dai et al. found that *Streptococcus* abundance was positively associated with glutathione, cysteine, and methionine levels, and that the activation of these metabolic pathways was increased in gastric cancer [228].

Oral microbiota can metabolize alcohol (ethanol) to acetaldehyde due to the presence of the enzyme alcohol dehydrogenase (ADH), which is involved in carcinogenesis. Several species of oral bacteria, such as *S. mitis*, *S. gordonii*, *S. salivarius*, *S. sanguinis*, and *S. oralis*, possess ADH, which metabolizes alcohol to acetaldehyde, a compound with carcinogenic potential [99].

#### 3.4.6. Pancreatic Cancer

*P. gingivalis* stimulates proteinase-activated receptor 2 (PAR2) and then activates the pathway of PAR2/NF-κB. Meanwhile, *P. gingivalis* can activate ERK1/2-Ets1 and p38/HSP27 pathways after invading host cells. The above three pathways jointly induce the expression of proMMP-9. Activated MMP-9 can degrade a variety of extracellular matrix (ECM) components through proteolytic cleavage. Destruction of the ECM is a necessary step during tumor invasion and metastasis, and studies have shown that MMP-9 is overexpressed in PDAC [266].

## 4. Conclusions

An increasing body of evidence indicates that the oral microbiota exerts a significant influence on carcinogenesis, extending beyond the oral cavity to distant organs. Pathogens such as *P. gingivalis*, the *F. nucleatum* group, and *T. denticola* contribute to cancer development through multiple mechanisms, including chronic inflammation, direct genotoxicity, modulation of the immune response, and epigenetic alterations, thereby creating a tumor-promoting microenvironment.

The detection of oral bacteria in tumor tissues of the gastrointestinal, respiratory, and urogenital systems, as well as in the liver and pancreas, highlights the existence of an oral–systemic microbial axis that may serve as a potential risk factor for various malignancies. Although a direct causal relationship has not yet been definitively established, accumulating data strongly suggest that oral microbiota dysbiosis is an important cofactor in cancer development.

Understanding the role of oral pathogens in carcinogenesis opens new avenues for prevention, diagnosis, and treatment. In the future, microbiome-based biomarkers could become part of screening strategies for at-risk populations, while therapeutic interventions targeting the microbiome—such as antimicrobial approaches, probiotics, or microbiota transplantation—may help reduce cancer incidence and progression.

Given the complexity of interactions between the microbiota, immune system, and tumor microenvironment, further multidisciplinary research integrating microbiology, oncology, immunology, and genomics will be essential. Such an approach may ultimately provide deeper insights into cancer pathogenesis and foster the development of innovative microbiome-based therapeutic strategies.

## Figures and Tables

**Table 1:** Presence of oral bacteria in carcinoma or oral samples of patients with OSCC.

Study Sample	Sample Type	Oral Bacteria and Main Findings	Ref.
25 OSCC patients and 24 healthy controls	Saliva	*Streptococcus anginosus* (*S. anginosus*), *Veillonella parvula*, *Porphyromonas endodontalis*, and *Peptostreptococcus anaerobius* could contribute to OSCC	[16]
60 OSCC patients and 80 non-cancer controls	Saliva	*Peptostreptococcus*, *Fusobacterium*, *Alloprevotella*, and *Capnocytophaga* spp. more abundant in OSCC	[20]
45 OSCC patients and 229 OSCC-free patients	Saliva	*Capnocytophaga gingivalis*, *P. melaninogenica*, and *Streptococcus mitis* (*S. mitis*) elevated in the saliva of individuals with OSCC	[25]
125 OSCC cases, 124 cases of epithelial precursor lesions, and 127 controls	Saliva	*Parvimonas* spp. present only in OSCC samples compared to epithelial precursor lesions and in healthy sites	[27]
51 controls, 41 OSCC stage 1, 66 OSCC stages 2 and 3, and 90 OSCC stage 4 patients	Oral rinse (sterile saline)	*Fusobacterium periodonticum*, *Parvimonas micra*, *Streptococcus constellatus*, *Haemophilus influenzae*, and *Filifactor alocis* associated with OSCC, progressively increased in abundance from stage 1 to stage 4	[28]
22 OSCC patients, 8 precancer patients, And 6 healthy individuals	Oral mucosal swabs	*Fusobacterium* significantly present in OSCC compared to contralateral healthy site	[26]
50 patients—50 paired samples were obtained from non-tumor (50) and tumor sites (50)	Swab	*F. nucleatum* group, *P. intermedia*, *Aggregatibacter segnis*, *Capnocytophaga leadbetteri*, and *Peptostreptococcus stomatis* significantly increased in OSCC	[21]
40 OSCC patients, 40 controls	Oral mucosal swabs	*Mycoplasma*, *Treponema*, *Campylobacter*, *Eikenella*, *Centipeda*, *Lachnospiraceae_G_7*, *Alloprevotella*, *Fusobacterium*, *Selenomonas*, *Dialister*, *Peptostreptococcus*, *Filifactor*, *Peptococcus*, *Catonella*, *Parvimonas*, and *Capnocytophaga* were more abundant in OSCC	[29]
20 OSCC samples and 20 deep-epithelium control swabs	OSCC tissue and swab	*F. polymorphum* (*F. nucleatum* group) was the most significantly overrepresented species in the tumors, followed by *Pseudomonas aeruginosa* (*P. aeruginosa*) and *Campylobacter* spp.	[30]
42 OSCC, 2 lymphoma, 2 rhabdomyosarcoma, and 3 leukoplakia	Tissue	*S. anginosus* present in OSCC (19/42) and not in the other type of oral cancers nor in the leukoplakia	[17]
25 OSCC patients and 27 patients with fibroepithelial polyp	Tissue	Genera *Capnocytophaga*, *Pseudomonas*, and *Atopobium* associated with OSCC	[18]
10 OSCC and 5 normal gingiva samples	Paraffin-embedded gingival tissue	Higher levels of *P. gingivalis* (more than 33%) detected in OSCC samples	[19]
169 patients with paired adjacent OSCC and control tissue	Tissue	*Parvimonas* sp. was increased in OSCC samples	[23]
20 tissue samples (10 OSCC samples and 10 non-tumor samples)	Tissue	*Streptococcus* sp. oral taxon 058, *Streptococcus salivarius* (*S. salivarius*), *Streptococcus gordonii*, *Streptococcus parasanguinis*, *Peptostreptococcus stomatis*, *Gemella haemolysans*, *Gemella morbillorum*, and *Johnsonella ignava* increased in abundance in tumor samples	[24]
20 OSCC patients and 12 control tissues	Tissue	*Exiguobacterium oxidotolerans*, *P. melaninogenica*, *Staphylococcus aureus*, *Veillonella parvula*, and *Micrococcus luteus* detected in OSCC, not in controls	[31]
10 OSCC patients and the same patient controls	Tissue	*Fusobacterium naviforme* present in OSCC samples, not in nontumorous tissue	[32]
61 OSCC patients and 30 controls	Tissue	*P. gingivalis* and *Fusobacterium* increased in OSCC	[33]
24 OSCC patients and 24 controls	OSCC tissue and brush biopsy controls	*P. intermedia* and *P. gingivalis* frequently detected in OSCC samples	[34]

**Table 2:** Presence of oral bacteria in carcinoma or oral samples of patients with esophageal cancer.

Study Sample	Sample Type	Oral Bacteria and Main Findings	Ref.
EC, *n* = 39 Control, *n* = 51	Saliva	*Neisseria*, *Prevotella*, and *Veillonella* potential new biomarkers for EC	[47]
ESCC, *n* = 32 Control, *n* = 35	Saliva	At the phylum level, in ESCC patients, there were comparatively greater amounts of Firmicutes and Bacteroidetes (25.3% vs. 24.9%) and lower amounts of Proteobacteria; at the genus level, ESCC patients exhibited comparatively greater amounts of *Streptococcus* spp. and *Prevotella* spp. than healthy controls	[44]
ESCC, *n* = 90 Control, *n* = 50	Saliva	*Leptotrichia* spp., *Fusobacterium* spp., *P. gingivalis*, and *S. salivarius* were more abundant in ESCC patient saliva than in healthy controls’ saliva	[45]
ESCC, *n* = 87 Control, *n* = 85	Saliva	*Prevotella* spp., *Streptococcus* spp., *Fusobacterium* spp., and *Veillonella* spp. were the most predominant genera in the ESCC group	[48]
EAC, *n* = 81 Matched controls, *n* = 160 ESCC, *n* = 25 Matched controls, *n* = 50	Mouthwash samples	* T. forsythia * is associated with a higher risk of EAC; genus *Neisseria* and the species *Streptococcus pneumoniae* were associated with lower EAC risk; and *P. gingivalis* trended with a higher risk of ESCC	[49]
ESCC, *n* = 61 Control, *n* = 62	Oral swabs	The prevalence of *T. forsythia*, *S. anginosus*, *A. actinomycetemcomitans*, and *F. nucleatum* group were associated with a high risk of ESCC	[50]
ESCC, *n* = 34 Control, *n* = 18	Oral biofilm	*P. gingivalis*, *Veillonella* spp., and *Streptococcus* spp. had higher abundance in patients with ESCC	[51]
ESCC, *n* = 66 Control, *n* = 67	Tumor biopsy	ESCC tumor tissues contained more *Fusobacterium* than nontumor tissues	[43]
ESCC, *n* = 17 Control, *n* = 16	Tumor biopsy	ESCC specimens were rich in *Fusobacterium* spp., *Prevotella* spp.	[41]
ESCC, *n* = 32 Control, *n* = 21	Tumor biopsy	Greater abundance of *Streptococcus* spp., *Actinobacillus* spp., *Peptostreptococcus* spp., *Prevotella* spp., and *Fusobacterium* spp. than healthy controls	[40]
ESCC, *n* = 100 Control, *n* = 100	Tumor biopsy	*P. gingivalis* was detected immunohistochemically in 61% of cancerous tissues and was undetected in normal esophageal mucosa	[46]
ESCC, *n* = 45 Without control	Tumor biopsy	Bacteroidetes, Firmicutes, and Spirochaetes have significantly higher relative abundances among positive lymph node patients; the abundance of only *Streptococcus* spp. in stage 3 and 4 was significantly higher than that in stages 1 and 2; and combined *Streptococcus* spp. and *Prevotella* spp. abundance associated with unfavorable survival	[52]
ESCC, *n* = 111 Control, *n* = 41 (normal tissues)	Tumor biopsy	Relative abundances of *Fusobacterium* spp. and *Prevotella* spp. were correlated with clinical stage in ESCC, where they were higher in tumors than in the corresponding normal tissues	[53]

## Data Availability

Not applicable.
